# Impact of clinical clerkship integrated with clinical ladder on attending physicians’ teaching self-efficacy

**DOI:** 10.1186/s12909-024-05396-0

**Published:** 2024-04-10

**Authors:** Yuto Arai, Go Yoshino, Kento Ohta, Tohru Okanishi, Sosuke Kakee, Yoichi Mino, Hiroaki Komatsu, Nanako Yamada, Masaru Ueki, Yoshihiro Maegaki

**Affiliations:** 1 Division of Child Neurology, Department of Brain and Neurosciences, Faculty of Medicine, Tottori University, 36-1 Nishi-Cho, Yonago, Tottori 683-8504 Japan; 2 Division of Pediatrics and Perinatology, Department of Multidisciplinary Internal Medicine, School of Medicine, Tottori University Faculty of Medicine, Yonago, Japan; 3 Department of Obstetrics and Gynecology, Tottori University School of Medicine, Yonago, Japan; 4 Center for Clinical Residency Program, Tottori University Hospital, Yonago, Japan; 5 Division of Medical Education, School of Medicine, Faculty of Medicine, Tottori University, Yonago, Japan

**Keywords:** Clinical teaching, Self-efficacy, Teaching involvement, Teaching quality, Undergraduate medial education, Clinical ladder program

## Abstract

**Background:**

Self-efficacy plays an important role in enhancing the teaching capabilities of attending physicians (APs). The clinical ladder (CL) is an educational approach developed in the field of nursing education that increases difficulty in an incremental manner. However, no previous study has confirmed the effectiveness of CL in medical education. Therefore, this study aimed to examine the effect of clinical clerkship integrated with clinical ladder (CC-CL) on the self-efficacy of APs.

**Methods:**

Sixth-year medical students participated in CC-CL for 6 months starting from April 2023, and the changes in the self-efficacy of APs were retrospectively evaluated. The students were trained by the APs concurrently, and the achievement levels of each student were shared. The primary outcome measure was the physician teaching self-efficacy questionnaire (PTSQ) score. The PTSQ scores before and after CC-CL were analyzed using the Wilcoxon matched-pair signed-rank test.

**Results:**

Fifteen APs from the Department of Pediatric and Child Neurology were included in this study. No significant difference was observed in the total PTSQ scores of the APs before and after CC-CL. However, a significant increase was observed in the PTSQ score of APs who participated for at least 2 h per week over a period of more than 3 months (*n* = 8) after CC-CL (*p* = 0.022). Furthermore, APs who had received their pediatrician certification < 10 years ago (*n* = 8) showed a significant increase in the total PTSQ score after CC-CL (*p* = 0.022).

**Conclusions:**

CC-CL may play an important role in cultivating the self-efficacy of less experienced APs. Further comparative studies must be conducted in the future to validate the findings of this study.

**Supplementary Information:**

The online version contains supplementary material available at 10.1186/s12909-024-05396-0.

## Background

Teaching self-efficacy refers to the confidence and belief of medical teachers in their ability to provide excellent instruction [1, 2]. Self-efficacy influences the teaching quality over time [1, 3, 4]. Confident teachers demonstrate higher job satisfaction [5, 6], better well-being [7–9], greater commitment [10, 11], and effectiveness [12]. Teachers’ confidence in teaching has been shown to boost academic achievement [13–15] and motivation [16] among students. Thus, increasing the self-efficacy of medical teachers would benefit both teachers and students [17].

Clinical clerkship (CC) or rotation refers to the practice of medicine by medical students during their fifth and sixth years of study [18]. CC comprises assessing the motivation, skills, and knowledge attainment levels of each student and assigning advanced roles as their abilities improve [19]. However, the shortage of attending physicians (APs) and their heavy workload has affected CC in Japan [20]. Consequently, the Ministry of Education, Culture, Sports, Science and Technology of Japan has aimed to strengthen the collaborative framework among APs and enhance their educational skills [21]. However, the educational system in CC varies among facilities and remains to be established.

The clinical ladder (CL) is an educational approach developed in the field of nursing to foster excellence in bedside nursing [22, 23], wherein levels are assigned based on the difficulties encountered in the clinical setting. The CL encompasses three levels: entry-level, requiring basic nursing skills and knowledge; intermediate-level, involving complex patient care and leadership roles; and advanced-level, requiring specialized knowledge and involvement in educational program development. The number of these steps may vary depending on the country or hospital [24]. CL gradually equips individuals with advanced skills. Thus, incorporating CL into CC will facilitate the gradual assignment of tasks to medical students and foster collaboration among APs by sharing the achievement levels of each student. Consequently, it is anticipated that balanced educational opportunities could be provided to APs, thereby enhancing their educational skills. This study aimed to examine the impact of CC integrated with CL (CC-CL) on the self-efficacy of APs.

## Methods

### Participants

APs from the Department of Pediatric and Child Neurology at the Tottori University Faculty of Medicine Hospital were included in this study.

### Intervention

**Fig. 1:**
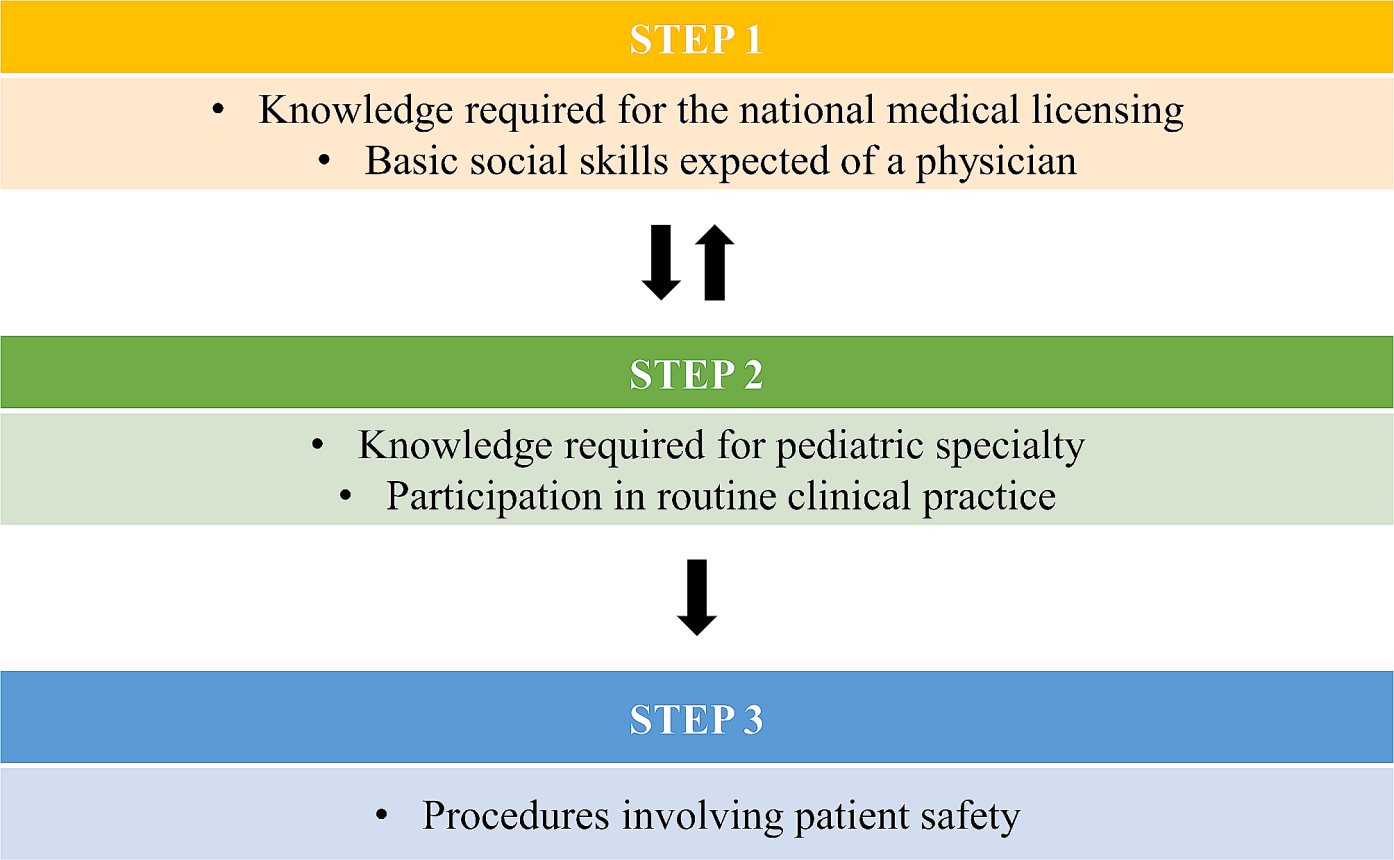
The clinical ladder created by the attending physicians. Students carry this during their clinical rotations. Attending physicians verify the achievements of the students and sign off on them. Achievement goals are individually set based on the motivation and abilities of the students

The levels of achievement within CC-CL were divided into three steps, with APs tasked to educate students in accordance with these steps (Fig. 1). In this integration of CC and CL, STEP 1 refers to possessing the knowledge required for the national medical licensing examination (e.g., knowledge related to Apgar scores) and basic social skills expected from a physician (e.g., greeting healthcare staff). STEP 2 refers to possessing the knowledge required for pediatric specialty training (e.g., knowledge related to initial responses to anaphylaxis) and responsibilities in routine clinical practice (e.g., managing inpatient care with APs). STEP 3 focuses on procedures involving patient safety (e.g., blood collection and IV insertion). A checklist of approximately ten items was established for each step, and the accomplishments of the students were assessed periodically using the checklist of accomplishments (Supplementary Table 1). Evaluations were conducted by specific APs, as well as all APs in the department. The APs progressed to STEP 3 after completing STEPs 1 and 2 (STEP 1 and STEP 2 are interchangeable), while adjusting the achievement goals based on the motivation, skills, and knowledge attainment levels of the students. For example, APs expected students aspiring to become pediatricians to reach STEP 3, while those aiming for basic pediatric medical skills were expected to reach STEP 2.

### Data analysis

#### Attending physicians

The physician teaching self-efficacy questionnaire (PTSQ) was developed by Dybowski et al. to evaluate the self-efficacy of teaching in a medical setting [2]. The PTSQ comprises the following three subscales. First, self-regulation encompasses items addressing the obstacles encountered by instructors while teaching. A sample self-regulation item is “Even if students ask difficult questions, I am able to answer them correctly.” Second, dyadic regulation comprises the management of the difficulties inherent in the teacher-student relationship. A sample dyadic regulation item is “Even if students seem tired or demotivated, I manage to make them enthusiastic about the lesson.” Third, triadic regulation encompasses the management of the challenges arising from the interactions among teachers, students, and patients. A sample triadic regulation is “I am a very good role model for the students in dealing with patients.” The responses are assessed using a five-point Likert scale, ranging from 1 to 5, with higher scores indicating higher levels of teaching self-efficacy. The PTSQ was translated into Japanese by YA and NY, who are proficient in English. The original and translated versions of PTSQ are provided in Supplementary Tables 2 and 3, respectively.

The self-reports of the APs were retrospectively analyzed. The primary outcome measure was the PTSQ scores of the physicians before and after CC-CL. Participants were instructed to answer questions regarding sex, years of experience, the duration/time of involvement in CC-CL, and the time spent on CC-CL per week to measure the basic attributes. Differences in PTSQ scores before and after participation in CC-CL were analyzed. Additionally, the differences in PTSQ scores were examined based on the number of years of experience and duration of time engaged in CC-CL. Finally, feedback on CC-CL was collected from APs using a questionnaire, with participation being voluntary.

#### Students

Sixth-year (final year) medical students at the Tottori University were selected to participate in CC. The students rotated through six clinical departments, spending one month in each for a total of six months. The students set their own achievement goals based on advice from APs. Some aimed to progress to STEP 3, while others set different goals, such as achieving 80% for STEP 1 and 50% for STEP 2. After receiving feedback from APs, students obtained confirmation signatures and proceeded to the next achievement goal.

We conducted a satisfaction survey with students after CC-CL, gathering feedback on their satisfaction using a 5-point scale (1 = very dissatisfied; 5, very satisfied). In addition, the level of enthusiasm for APs was evaluated using another 5-point scale (1 = not at all enthusiastic; 5 = extremely enthusiastic).

### Statistical analysis

A Wilcoxon matched-pair signed-rank test was used to perform the analysis. A *p*-value of < 0.05 was considered statistically significant. Data analyses were performed using SPSS (version 25.0; IBM Japan, Tokyo, Japan).

### Ethical considerations

This study was approved by the Institutional Ethics Committee of the Tottori University Hospital (approval number: 23A128).

## Results

Questionnaires were collected from 25 APs before initiating CC-CL, and from 15 of these 25 APs after completion of CC-CL (*n* = 15). Table 1 presents the characteristics of. Twelve (80%) participants were males. One (6.7%) participant was a pediatric resident, whereas 14 (93.3%) were pediatricians certified as specialists. Eleven participants (73.3%) participated in CC-CL for > 3 months. Moreover, eight APs (53.3%) participated in CC-CL for > 2 h per week. Furthermore, among the 27 students participating in CC-CL, 21 (77.8%) rated CC-CL as “5” (very satisfied), and 25 students (92.6%) assigned the APs a rating of “5” (extremely enthusiastic) (Supplementary Table 4).

**Table 1:** Characteristics of participants

		*n* = 15
		n (%)
Sex	Male	12 (80.0%)
Years of experience	Pediatric resident	1 (6.7%)
	Pediatric specialization obtained less than 5 years ago	5 (33.3%)
	Pediatric specialization obtained between 5 and 10 years ago	2 (13.3%)
	Pediatric specialization obtained 10 or more years ago	7 (46.7%)
Duration involved in CC-CL (months)	<3	4 (26.7%)
	Between 3 and 6 months	2 (13.3%)
	6	9 (60.0%)
Time spent on CC-CL per week (hours)	<2	7 (46.7%)
	at least 2 h but less than 5 h	5 (33.3%)
	at least 5 h but less than 10 h	3 (20.0%)

CC-CL: clinical clerkships integrated with clinical ladder

### Changes in the total PTSQ score

No significant difference was observed in the total PTSQ score before and after CC-CL among all APs (*n* = 15) (Table 2). However, APs who engaged in CC-CL for > 3 months and 2 h per week (*n* = 8) showed a significant increase in the total PTSQ after CC-CL (Pre: 34.5 vs. Post: 45; *p* = 0.022). Furthermore, after participating in CC-CL, a significant increase was observed in the total PTSQ score of APs who obtained their pediatric certification < 10 years ago (*n* = 8; Pre: 30.5 vs. Post: 41; *p* = 0.022). Five participants (33.3%) met both APs who engaged in CC-CL for > 3 months and 2 h per week and APs who obtained their pediatric certification < 10 years ago.

**Table 2:** The changes in the total PTSQ scores and the three sub-scales before and after CC-CL

	Pre	Post	p-value
	Median		
**Total**			
All (*n*=15)	33	39	0.068
Involvement for more than 3 months (*n*= 11)	33	44	0.053
Involvement for more than 3 months and 2 h per week (*n*= 8)	34.5	45	0.022*
Pediatrician certification obtained <10 years ago (*n*= 8)	30.5	41	0.022*
Pediatrician certification obtained 10 or more years ago (*n*= 7)	36	39	1
**Self-regulation self-efficacy**			
All (*n*=15)	15	16	0.15
Involvement for more than 3 months (*n*= 11)	15	17	0.17
Involvement for more than 3 months and 2 h per week (*n*= 8)	15	17.5	0.11
Pediatrician certification obtained <10 years ago (*n*= 8)	14	16.5	0.073
Pediatrician certification obtained 10 or more years ago (*n*= 7)	15	16	1
**Dyadic regulation self-efficacy**			
All (*n*=15)	8	11	0.12
Involvement for more than 3 months (*n*= 11)	8	12	0.065
Involvement for more than 3 months and 2 h per week (*n*= 8)	9	13	0.021*
Pediatrician certification obtained <10 years ago (*n*= 8)	7	11.5	0.034*
Pediatrician certification obtained 10 or more years ago (*n*= 7)	10	9	0.78
**Triadic regulation self-efficacy**			
All (*n*=15)	11	12	0.14
Involvement for more than 3 months (*n*= 11)	11	12	0.14
Involvement for more than 3 months and 2 h per week (*n*= 8)	11.5	12.5	0.14
Pediatrician certification obtained <10 years ago (*n*= 8)	10.5	12.5	0.11
Pediatrician certification obtained 10 or more years ago (*n*= 7)	11	11	0.93

PTSQ: Physician teaching self-efficacy questionnaire; CC-CL: clinical clerkship integrated with clinical ladder; *: *p*<0.05

### Changes in the PTSQ subscale

No significant differences were observed in the three subscales across all APs. However, dyadic regulation of self-efficacy increased significantly in APs who engaged in CC-CL for > 3 months and 2 h per week (Pre: 9 vs. Post: 13; *p* = 0.021) and in those who obtained their pediatric certification < 10 years ago (Pre: 7 vs. Post: 11.5; *p* = 0.034).

### Feedback from APs

We gathered feedback via a questionnaire survey (Supplementary Table 5), including responses from APs who engaged in CC-CL for > 3 months and 2 h per week, as well as from those who obtained their pediatric certification < 10 years ago. Some APs expressed positive opinions regarding the clarification provided for teaching objectives, while some believed that CC-CL influenced students’ motivation and engagement.

## Discussion

The evaluation of the effect of CC-CL on the self-efficacy of APs revealed no significant effect in the present study. However, self-efficacy, especially in the student-physician relationship, was significantly different before and after CC-CL among APs certified as specialists < 10 years ago and APs engaged for more than 2 h a week for more than 3 months.

CC-CL improved the self-efficacy of APs who expended a certain amount of effort toward CC-CL. The students’ tasks were clarified and hierarchized to make them attainable. Furthermore, individual goals were tailored to each student’s motivation, skills, and knowledge level, which also proved beneficial for the APs. Achieving goals through teaching is the most influential source of self-efficacy [12], and educators with high self-efficacy tend to expend more effort in education [25]. Moreover, cooperation and communication within a team can influence the self-efficacy of an individual [26, 27]. Therefore, the awareness of teaching as a team rather than individually, coupled with the sense of accomplishment derived from increased educational opportunities, may have contributed to the enhanced self-efficacy of the APs.

CC-CL increased the self-efficacy of less-experienced APs. Self-efficacy is a highly valuable factor that plays a crucial role in enhancing job satisfaction. Moreover, it has a preventive effect against burnout, which is defined as emotional exhaustion, depersonalization, and a decreased sense of personal accomplishment owing to work-related stress [28–32]. Burnout is a significant issue affecting residents and APs [32–35] and has been associated with absenteeism, high workplace turnover, and decreased job satisfaction [36–38]. Therefore, preventing burnout among APs through CC-CL could potentially improve the working environment.

This study has several limitations. First, the reliability of the Japanese version of PTSQ was not validated, which may have led to under- or overestimation of teaching self-efficacy. However, positive evaluations of CC-CL from APs suggest a potential positive impact on them. Second, this was a small-scale, single-center study. Furthermore, the effect of CC-CL on medical students was not examined. Nonetheless, given that teachers’ high self-efficacy is known to enhance students’ academic performance and motivation [13–16], continuing CC-CL may also have potential positive impact on students.

## Conclusions

CC-CL may play an important role in cultivating self-efficacy in less experienced APs who expend a certain amount of effort into CC-CL. Given the high level of student satisfaction, CC-CL could be considered a potential option for establishing an efficient educational system in clinical clerkship. Further comparative studies must be conducted in the future to validate these findings.

### Electronic supplementary material

Below is the link to the electronic supplementary material.

Supplementary Material 1

Supplementary Material 2

Supplementary Material 3

Supplementary Material 4

Supplementary Material 5

## Data Availability

The data supporting the findings of this study are available from the corresponding author upon reasonable request.

## Abbreviations

CC: Clinical clerkship
AP: Attending physician
CL: Clinical ladder
CC-CL: Clinical clerkship integrated with clinical ladder
PTSQ: Physician teaching self-efficacy questionnaire
